# Evaluating the Effectiveness of Culturally Adapted Persian-Translated PEERS® (Program for the Education and Enrichment of Relational Skills) for Young Adults with ASD : A Mixed-Methods Study

**DOI:** 10.1192/j.eurpsy.2025.409

**Published:** 2025-08-26

**Authors:** M. Motamed, J. Alaghband-rad, S. Jamaloo, N. Tavakkolian, R. Amin, N. Khosravanmehr

**Affiliations:** 1 Psychiatry, Tehran University of Medical Sciences; 2 Psychiatry, Iran University of Medical Sciences, Tehran, Iran, Islamic Republic Of

## Abstract

**Introduction:**

Social deficits are a significant impairment in adult ASD, leading to difficulties in socio-emotional cognition, communication, and relationships. Effective social skills interventions are crucial during the transition to adulthood. The UCLA PEERS® (Program for the Education and Enrichment of Relational Skills) for young adults is a prominent and well-researched intervention, implemented in over eighty countries and translated into numerous languages. This program includes concurrent sessions for young adults and their parents, aiming to improve social skills and reduce ASD-related social issues

**Objectives:**

The current study aimed to test the effectiveness of a culturally adapted Persian -translated version of PEERS® for young adults with ASD without intellectual disabilities.

**Methods:**

Three psychiatrists and two psychologists underwent PEERS® training and translated the manual into Farsi, adapting it culturally for Iran. Twenty-four young adults with ASD and their parents participated in the study, randomly assigned to a treatment group or a control group. Inclusion criteria were males aged 18-30 years with an ASD diagnosis, predominant social difficulties, motivation to participate, and a family member acting as a social coach. Exclusion criteria included intellectual disability, major psychiatric conditions, and significant verbal or literacy difficulties.

Participants attended 16 weekly 90-minute group sessions, covering topics like conversations, humor, electronic communication, dating etiquette, conflict management, and bullying. Sessions were led by a PEERS-certified psychologist and an assistant behavioral coach. Weekly meetings were held to align session content and address potential issues.

**Results:**

The culturally adapted Persian -translated version of PEERS® was perceived as beneficial by young adults with ASD and their caregivers, improving social skills and quality of life. However, quantitative measures did not show significant changes, indicating a need for further refinement and investigation to achieve measurable improvements in social functioning.

**Conclusions:**

While qualitative feedback indicates that both young adults with ASD and their caregivers found the caregiver-assisted social skills intervention to be beneficial in improving social skills and overall quality of life, the quantitative measures did not show significant changes. This suggests that while the intervention may have positive perceptual impacts, further refinement and investigation are needed to achieve measurable improvements in social functioning.

**Disclosure of Interest:**

None Declared

